# Nature-Identical Safranal and Dihydrocoumarin from *Ageratina adenophora* ((Spreng., 1970) King and H. Rob.) Target Energy Metabolism to Control *Solenopsis invicta* Buren, 1972 (Hymenoptera: Formicidae)

**DOI:** 10.3390/insects16050540

**Published:** 2025-05-20

**Authors:** Mingqi Wu, Rongchao Luo, Mehboob Hussain, Wenmei Wu, Shini Li, Zijun Guo, Boyu Jia, Gaofeng Bi, Xi Gao, Guoxing Wu, Deqiang Qin

**Affiliations:** 1 State Key Laboratory for Conservation and Utilization of Bio-Resources in Yunnan, Yunnan Agricultural University, Kunming 650201, China; 18314326424@163.com (M.W.); 13097482720@163.com (R.L.); mehboobhussain413@gmail.com (M.H.); lishini06@163.com (S.L.); ynau_gzj@126.com (Z.G.); boyuhhh@outlook.com (B.J.); 15308753937@163.com (G.B.); chonchon@163.com (X.G.); 2 School of Life Sciences and Biopharmaceutics, Guangdong Pharmaceutical University, Guangzhou 510006, China; wuwenmei@gdpu.edu.cn

**Keywords:** *Solenopsis invicta*, *Ageratina adenophora*, toxicity, insecticidal mechanism, secondary metabolites

## Abstract

The bio-prospecting of bioactive phytochemicals from invasive flora presents a sustainable paradigm for the ecologically conscious management of major invasive pest *S. invicta*. *Ageratina adenophora*, while recognized for its allelopathic insecticidal properties, exhibits poorly characterized toxicological profiles and mechanistic underpinnings against *S. invicta*, warranting systematic investigation to elucidate its mode of action. This study elucidates the bioactive insecticidal compounds of *A. adenophora* and their toxicological impacts on *S. invicta*, including behavioral, metabolic, and enzymatic perturbations, via liquid chromatography–mass spectrometry (LC-MS) profiling. The ethanol extracts of the roots, stems, and leaves of *A. adenophora* have shown control effects on *S. invicta*, with an LC_50_ (50% lethal concentration) of 331.847, 188.256, and 166.253 mg/mL at 48 h, respectively. Metabolite profiling of *A. adenophora* revealed that safranal and dihydrocoumarin are relatively high in plant leaves, and they showed significant insecticidal activity and behavioral inhibitory effects on *S. invicta* with LC_50_ 349.042 mg/L and 118.336 mg/L at 48 h, respectively. Notably, these two bioactive compounds disrupted the normal energy production through glucose metabolism and the citrate cycle, which eventually led to the death of *S. invicta*. Further, these two compounds also activated the detoxification metabolic pathway of *S. invicta*. These findings provide a theoretical basis for the use of these bioactive compounds in the integrated management of *S. invicta* and may lead to the development of a new biopesticide.

## Simple Summary

This study explored the ecological management strategy of developing bioactive compounds from the invasive plant *Ageratina adenophora* for the sustainable prevention and control of *Solenopsis invicta*. Through liquid chromatography–mass spectrometry analysis, the study found that the ethanol extracts of the roots, stems, and leaves of the plant had a dose-dependent insecticidal effect on *Solenopsis invicta*, among which the leaf extract had the most significant effect. Metabolomics identification showed that the leaves were rich in two active ingredients, safranal and dihydrocoumarin, which can effectively interfere with the behavior of ants and induce death mechanisms. These two compounds hinder energy production by disrupting glucose metabolism and the citric acid cycle while activating the pest detoxification metabolic pathway. This discovery provides a theoretical basis for the development of new biopesticides based on plant secondary metabolites, which will help establish a comprehensive prevention and control system for red fire ants.

## 1. Introduction

The red imported fire ant, *Solenopsis invicta* Buren (order: Hymenoptera; family: Formicidae), belongs to subfamily Myrmicinae and genus *Solenopsis* [1]. With over 200 species globally, including *Solenopsis richteri* and *Solenopsis geminata*, *S. invicta* stands out as an invasive threat [1,2]. First identified in South America’s Paraná River Basin, it has since spread globally via international trade, including to China, where it competes with native species like *Solenopsis chinensis* [3]. Its ecological aggression, rapid reproduction (queens lay >4500 eggs daily), and territorial defense mechanisms—mobilizing workers via pheromones to attack intruders—pose significant risks to biodiversity, agriculture, and human health [4]. Each ant possesses a terminal abdominal sting capable of injecting venom repeatedly, causing reactions ranging from localized inflammation to fatal anaphylaxis [5]. Effective management requires pesticides that induce high mortality while disrupting colony-wide behaviors [6].

Current control strategies for *S. invicta* prioritize chemical interventions (e.g., fipronil, indoxacarb, hydrazones) due to their immediate efficacy [7,8]. However, continuous excessive use of synthetic insecticides considerably increases insect resistance and tolerance and is inevitably harmful to non-target organisms and even humans [6,9]. Consequently, botanical alternatives based on biodegradable, low-toxicity compounds derived from plants are gaining attention. Extracts from pyrethrum, rotenone, and *Sophora flavescens* (matrine) show promise against *S. invicta* [10,11]. Notably, *Ageratina adenophora* (Asteraceae) exhibits insecticidal properties against pests like aphids and diamondback moths, but its activity against *S. invicta* remains uncharacterized. Critically, *A. adenophora* acts primarily as a stomach poison upon ingestion, with limited or no contact toxicity [12,13]. This mode of action aligns with fire ant feeding behaviors, making it a compelling candidate for targeted biocontrol.

*A. adenophora* is a perennial herb or semi-shrub of the genus Ageratina in the Asteraceae family, native to Mexico [9]. *A. adenophora* extracts are insecticidal primarily through ingestion as a stomach poison, i.e., it is not, or has limited activity as, a contact insecticide. A previous study proposed that the extracts of *A. adenophora* have significant toxicity and repellent activity against the larvae of *Plutella xylostella* [13]. *A. adenophora* produces diverse bioactive compounds, including flavonoids, terpenes, and sesquiterpenes [12,14]. Among them, sesquiterpenes have high inhibitory activity against *Pythium myriotylum* and *Phytophthora capsici*. Moreover, studies have found that the volatile oils from its flowers demonstrate antifungal and antifeedant activity against pests like the red palm weevil [15]. While *A. adenophora* extracts and essential oils demonstrate insecticidal efficacy, its broad-spectrum bioactive compounds pose risks to non-target organisms. *A. adenophora* has become a globally invasive species, now established across Asia, Europe, Oceania, and Africa [16,17]. Its proliferation poses profound ecological and economic consequences, disrupting native ecosystems and agricultural and livestock systems and posing risks to human and animal health [16,17]. The plant’s aggressive invasion strategy enables it to form dense monocultures, which suppress the growth of indigenous flora and diminish local biodiversity [18,19]. As *A. adenophora* is a notorious invasive weed, and *S. invicta* is recognized as one of the 100 most destructive invasive species in the world [20]. Using *A. adenophora* to explore its bioactive compounds and metabolic mechanisms for killing *S. invicta* can not only improve the utilization value of *A. adenophora* but also provide new ideas regarding the prevention and control of *S. invicta*.

Related studies have shown that coumarin compounds are toxic to *Drosophila melanogaster* and mites and have good inhibitory effects on plant bacteria and fungi [21,22,23]. Monoterpenoid compounds are also important in pest management [24]. While coumarins and related phytochemicals have long been recognized for their insecticidal properties [25], the efficacy of *A. adenophora*-derived safranal and dihydrocoumarin against *S. invicta* remains unexplored. This study evaluates the efficacy of safranal and dihydrocoumarin, two principal components of *A. adenophora* extracts, against *S. invicta* workers. By elucidating their metabolic and behavioral impacts, we aim to advance eco-friendly alternatives to synthetic pesticides.

## 2. Materials and Methods

### 2.1. S. invicta Collection and Rearing

*S. invicta* were collected from the farmland (subtropical) of Yunnan Agricultural University (25°03′36.90″ N 102°42′1.45″ E, altitude: 1912.90 m), Kunming, China. Mature ant nests were randomly selected, and soil containing approximately 8000 eggs, 1500 larvae, 2000 pupae, and 50,000 workers was rapidly excavated using a shovel and transferred to 20 L plastic buckets. The inner walls of the buckets were coated with talcum powder to prevent ant escape.

Colonies were acclimatized in the laboratory until natural re-nesting occurred. The “water drop method” [26], a technique where controlled water introduction into the nest simulates flooding, prompting ants to evacuate, was then applied to safely relocate the colony into artificial breeding boxes (30 × 20 × 15 cm). Ants were maintained on a diet of 10% (*w*/*v*) honey water and ham sausage ad libitum. Laboratory conditions were standardized at 25 ± 2 °C and 60–80% relative humidity under a 12:12 h light:dark cycle [27].

### 2.2. Plant Materials and Preparation

*A. adenophora* specimens were collected from Yunnan Agricultural University, China. Surface debris was removed by rinsing with distilled water. Plant tissues were categorized into three groups: new leaves (5–8 cm from stem tips), old leaves (≤30 cm above roots), stems, and roots. Tissues were dried in a forced-air oven at 40 °C for 12 h, then pulverized using a grinder, and sieved through a 40-mesh screen.

For extraction, 10 g of each powdered tissue was combined with 100 mL of 95% ethanol (*v*/*v*) and subjected to ultrasonic-assisted extraction (100 Hz, 25 °C) for 1 h. The mixture was filtered through Whatman No. 1 paper, and the extraction process repeated twice. The filtrates from three replicates were pooled and concentrated under reduced pressure using a rotary evaporator (40 °C, 100 rpm), yielding a semi-solid paste. Each paste (yield quantified gravimetrically) was stored at 4 °C until further analysis.

### 2.3. Toxicity Determination of Ethanol Extracts of A. adenophora to S. invicta

A total of 1 g of each crude extract (new leaves, old leaves, stems, roots) from *A. adenophora* was diluted with 10% (*w*/*v*) honey water to generate a two-fold serial dilution series (25, 12.5, 6.25, 3.12, and 1.56 mg/mL). A negative control consisting of 10% (*w*/*v*) honey water supplemented with ethanol equivalent to the lowest plant extract concentration (1.56 mg/mL) was included to account for potential solvent effects. All test and control solutions were prepared fresh prior to experimentation. Workers were starved for 24 h to standardize feeding behavior. Fifty ants were transferred to a 500 mL glass beaker (12 × 13.2 cm) coated internally with talcum powder to prevent escape. Two-milliliter centrifuge tubes (2 × 2.5 cm) were filled with six treatment concentrations (25–1.56 mg/mL) and a control solution. Non-absorbent cotton wool (sterile, pharmaceutical-grade) was used to plug tube openings, enabling capillary access to solutions while preventing leakage. Treatments were assigned to experimental units (beakers) via a random number generator (R script v4.2.1) to eliminate spatial bias. Worker cohorts were allocated randomly to ensure equal representation of colony sub-groups. The experiment was performed in triplicate and mortality recorded after every 24 h over six days.

### 2.4. Effects of Ethanol Extracts from A. adenophora Leaves on the Behavior of S. invicta

The crude extracts of *A. adenophora* old leaves at concentrations of 3.12 mg/mL and 1.56 mg/mL were selected to explore their effects on the behavior of *S. invicta* workers. Aggression, climbing, and grasping abilities of *S. invicta* workers were assessed following established protocols with modifications [24,28].

1. Grasping ability: Evaluated as per Xing et al. (2022) [28], briefly, 50 workers were placed in a talcum-coated plastic cup. After 10 s of inactivity, the cup was inverted over paper for 5–8 s. Workers remaining inside were counted as lacking grasping ability.
  Grasping (%) = (P1/P2) × 100%
  where P1 = the number of workers with grasping ability and P2 = the total number of workers in the test.
2. Climbing ability: Adapted from Shan et al. (2022) [23], ants were coaxed onto a marked bamboo stick (20 cm long, 2 cm wide). Individuals climbing ≥3 cm were recorded as competent.
  Climbing (%) = (P1/P2) × 100%
  where P1 = the number of workers with climbing ability and P2 = the total number of workers in the test.
3. Aggression determination: We took a thin bamboo stick and touched the antennae of the workers so that the worker’s upper jaw tightly bit the bamboo stick. We lifted the bamboo stick 5 cm above the table. If it did not fall, it was considered to have aggressive ability.
  Attacking (%) = (P1/P2) × 100%
  where P1 = the number of workers with attacking ability and P2 = the total number of workers in the experiment.

All assays included three replicates (n = 50 workers per replicate) under randomized treatment assignments.

### 2.5. Detection of Bioactive Compounds from A. adenophora

#### 2.5.1. Sample Extraction

After the sample was freeze-dried by vacuum, 50 mg of the sample was weighed, 1000 μL of the extracting solution (methanol:acetonitrile:water volume ratio = 2:2:1) was added, and this mixture was vortexed for 30 s. Steel beads were added, and the sample was processed by 45 Hz grinding for 10 min and ultrasonicated for 10 min (ice water bath). The sample was left to stand at −20 °C for one hour, and it was centrifuged at 4 °C, 12,000 rpm for 15 min. Then, 500 μL of the supernatant was carefully taken out and placed in an EP tube, the extract was dried in a vacuum concentrator, 160 μL of extracting solution (acetonitrile water volume ratio: 1:1) was added to the dried metabolites for re-dissolution, and this mixture was vortexed for 30 s and ultrasonicated for 10 min in an ice water bath. The sample was centrifuged at 4 °C, 12,000 rpm for 15 min. A total of 120 μL of supernatant was carefully taken out and placed in a 2 mL injection bottle, and 10 μL of each sample was mixed into a QC (quality control) sample for machine testing.

#### 2.5.2. UPLC-MS Conditions

Positive ion mode: mobile phase A: 0.1% formic acid, 5 mM ammonium acetate aqueous solution; mobile phase B: 0.1% formic acid acetonitrile. Negative ion mode: mobile phase A: 0.1% formic acid, 5 mM ammonium acetate aqueous solution; mobile phase B: 0.1% formic acid acetonitrile. Injection volume: 2 μL. MS conditions: electrospray ionization (ESI) temperature 550 °C; ion spray voltage (IS) 5500 V (positive ion mode)/−4500 V (negative ion mode); ion source gas I (GSI), gas II (GSII), and curtain gas (CUR) were set to 50, 55, and 35 psi, respectively, and collision-induced ionization parameters were set to medium. Instrument tuning and mass calibration were performed using 10 and 100 μmol/L polypropylene glycol solutions in QQQ and LIT modes, respectively. MRM mode was used for QQQ scanning, and the collision gas (nitrogen) was set to medium. DP and CE of each MRM ion pair were completed by further optimization of declustering potential (DP) and collision energy (CE). A specific set of MRM ion pairs was monitored in each period according to the metabolites eluted in each period.

### 2.6. Toxicity Determination of Safranal and Dihydrocoumarin to S. invicta

Safranal and dihydrocoumarin were dissolved in ethanol and diluted with 10% (*w*/*v*) honey water to achieve final test concentrations of 500, 250, 125, 62.5, and 31.25 mg/mL. Ethanol concentrations in all solutions were maintained below 1% (*v*/*v*) to minimize solvent effects. For toxicity testing, starved *S. invicta* workers were exposed to these concentrations using the water test tube method. Controls received 10% honey water containing equivalent ethanol volumes. Assays were replicated three times with freshly prepared solutions.

### 2.7. Effects of Safranal and Dihydrocoumarin on the Metabolism of S. invicta

Workers of *S. invicta* were exposed to 48 h LC_50_ concentrations of safranal (Q) and dihydrocoumarin (RQ) for 48 h. Specimens, including moribund and surviving individuals, were collected post-treatment. Following exposure, specimens were flash-frozen in liquid nitrogen and submitted to Biomarker Technologies (Beijing, China) for non-targeted metabolomic profiling using ultra-performance liquid chromatography–tandem mass spectrometry (UPLC-MS/MS).

### 2.8. Effects of Safranal and Dihydrocoumarin on the Behavior of S. invicta

Aggression ability, climbing ability, and attack ability were measured according to the above methods as described in Section 2.4. When measuring walking, the workers were placed on a white paper with a 1 cm × 1 cm grid. If the workers could crawl 3 cm or more within 3 s, it was considered to have the ability to walk [29].

Walking = number of workers with walking ability/total number of workers × 100%

### 2.9. Effects of Safranal and Dihydrocoumarin on Enzyme Activities of S. invicta

The workers were fed with honey water containing the LC_50_ and double LC_50_ concentrations of safranal and dihydrocoumarin at 48 h, respectively. After 48 h of treatment, the activity changes of superoxide dismutase, insect cytochrome P-450 enzyme, glutathione S-transferase, and carboxylesterase in the workers were measured. The experiment was repeated three times.

Superoxide dismutase was measured using an ELISA reader according to the kit instructions of Beijing Box Biotechnology Co., Ltd. (Beijing, China).

$$\mathrm{SOD}\;(U/\mathrm{mL})=\frac{10\times Inhibition\;percentage\times D}{1-Inhibition\;percentage}$$

$$\mathrm{Inhibition}\;\mathrm{percentage}=\frac{\Delta Ablank-\Delta ATreatment}{\Delta Ablank}$$

where D: dilution multiple of crude enzyme solution.

Insect cytochrome P-450 enzyme was determined by using an ELISA reader according to the kit instructions of Jiangsu Jingmei Biotechnology Co., Ltd. (Yancheng, China). The calculation method was as follows: use the standard provided by the kit to make a standard curve with concentration as the horizontal axis and absorbance as the vertical axis and then use this to calculate the absorbance value of the experiment. Glutathione S-transferase was determined by using an ELISA reader according to the kit instructions.

GST (U/mL) = 0.23 ΔA

where ΔA: the difference between the absorbance value at 310 s and the absorbance value at 10 s in the assay group—the difference between the absorbance value at 310 s and the absorbance value at 10 s in the blank group.

Carboxylesterase was determined by using an ELISA reader according to the kit instructions.

CarE (U/mL) = 4 ΔA

where ΔA: the difference between the absorbance value at 310 s and the absorbance value at 10 s in the assay group—the difference between the absorbance value at 310 s and the absorbance value at 10 s in the blank group.

## 3. Data Analysis

All mortality data were expressed as means ± SE (standard error). Normality of the data was verified by the Shapiro–Wilks test, and the data were then analyzed using one-way ANOVA, followed by Duncan’s method to separate means. Differences were considered significant at the 0.05 significance level. All statistical analyses were performed using SPSS version 26.0 (SPSS Inc., Chicago, IL, USA). The mortality graphs were drawn using GraphPad Prism 10.5.1. For the metabolomics analysis, raw data were qualitatively and relatively quantitatively analyzed using the omics data processing software Progenesis QI v2.3, and the raw data were preprocessed for standardization. After screening, all peak signal intensities (peak areas) were normalized in sections, and peak identification and peak filtering were performed to correct the mass spectrometry peaks of the same metabolite in different samples. Principal component analysis (PCA) was used to analyze and distinguish the overall differences in metabolism among the groups and to screen differential metabolites. The structures of the compounds were inferred based on the accurate mass, secondary fragments, and isotope distribution using the PMDB (Plant Metabolome Database). Pathway enrichment analysis was performed using the KEGG (Kyoto Encyclopedia of Genes and Genomes) of differential metabolites to obtain metabolic pathway enrichment results. The hypergeometric test was applied to find metabolic pathway entries that were significantly enriched in the significantly differentially expressed metabolites compared with the entire background.

## 4. Results

### 4.1. LC-MS Analysis to Explore Bioactive Compounds from A. adenophora

**Figure 1:**
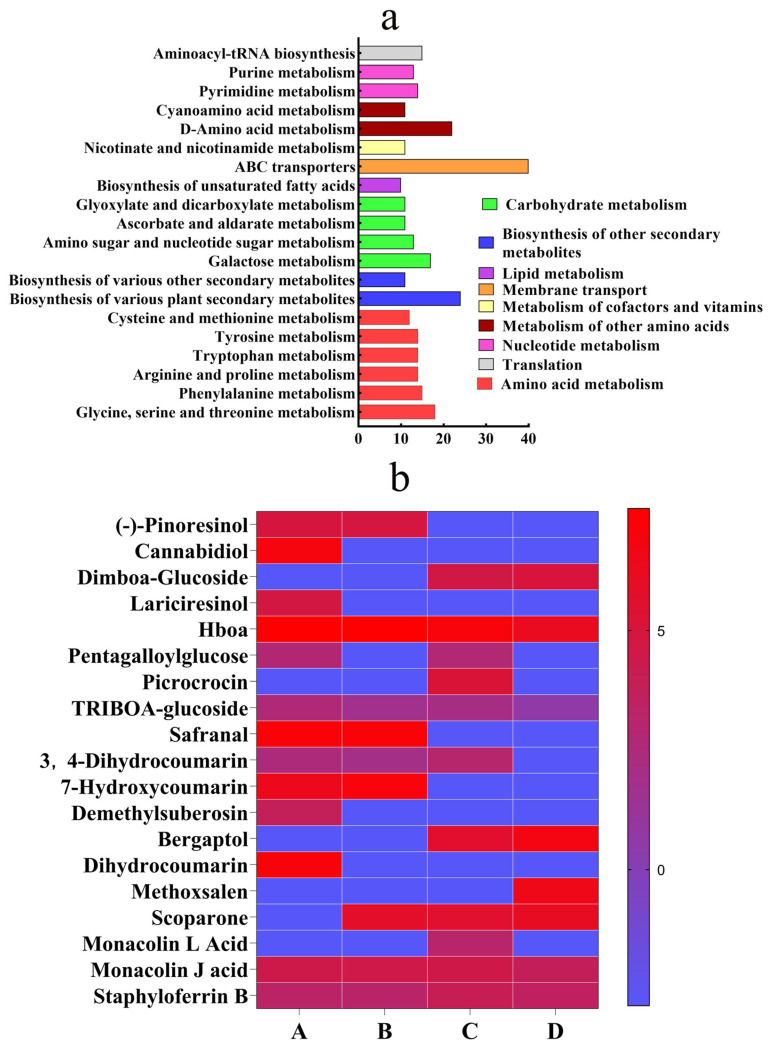
KEGG metabolite annotation and relative content heat map. The items under the same box in the figure represent the hierarchical classification annotation of the KEGG pathway, and the column length represents the number of metabolites annotated to the pathway. (**a**) KEGG database classification annotation. (**b**) Relative content of differential compounds in A (new leaves), B (old leaves), C (stems), and D (roots).

Metabolites were annotated using the KEGG database, with the top 20 enriched pathways mapped to level 3 KO entries. Identified metabolites were classified into nine principal pathways: amino acid metabolism, biosynthesis of other secondary metabolites, carbohydrate metabolism, lipid metabolism, membrane transport, metabolism of cofactors and vitamins, metabolism of other amino acids, nucleotide metabolism, and translation (Figure 1a). The biosynthesis of other secondary metabolite pathways contained 19 differentially abundant compounds (Figure 1b).

Safranal and dihydrocoumarin were selected for further insecticidal evaluation based on their high relative abundance in leaf extracts (Figure 1b) and prior evidence of their bioactivity against arthropods. Specifically, safranal is a known neurotoxic monoterpenoid in colchicine-based plant defenses, while dihydrocoumarin exhibits insect growth-regulatory properties through chitin synthesis inhibition.

### 4.2. Toxicity and Behavioral Effects of Ethanol Extracts of A. adenophora on S. invicta

**Figure 2:**
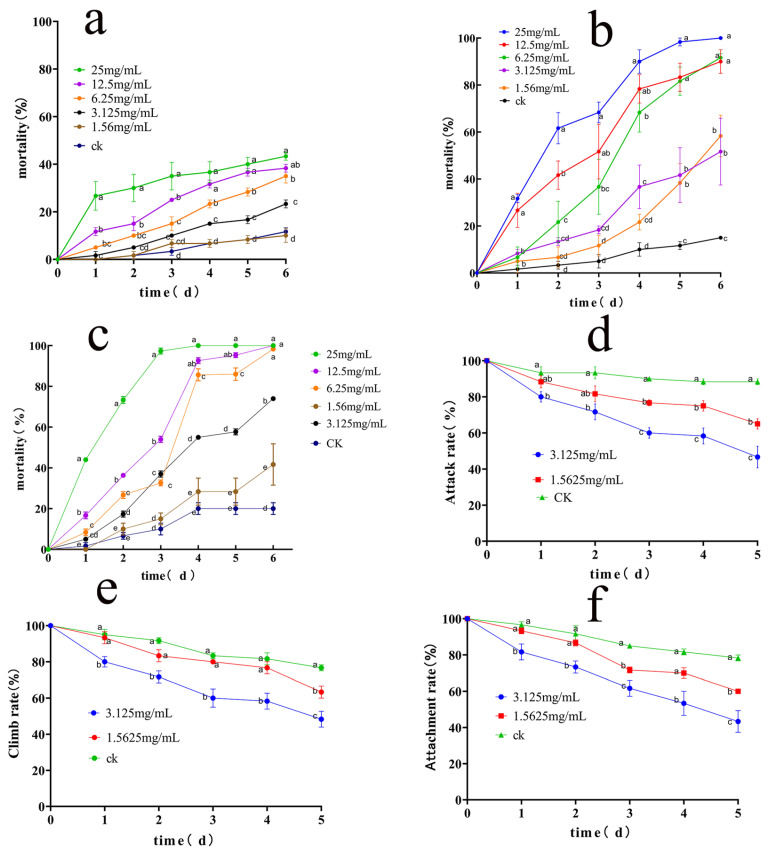
Toxicity and behavioral effects of ethanol extracts of *Ageratina adenophora* on *Solenopsis invicta*. (**a**–**c**) Toxicity of ethanol extracts of *Ageratina adenophora* root, stem, and leaf to *Solenopsis invicta*. (**d**–**f**) Effects of crude ethanol extracts from leaves on the behavior of *Solenopsis invicta*. Data are presented as mean ± standard error (S.E.). Different letters above bars indicate significant differences in mortality due to concentration effects within treatment at *p* < 0.05 level.

Ethanolic extracts from *A. adenophora* roots (LC_50_ = 331.84 mg/L), stems (LC_50_ = 188.25 mg/L), and leaves (LC_50_ = 166.25 mg/L) exhibited dose-dependent mortality in *S. invicta* workers (Figure 2a–c, Table 1). With the increase in concentration, the mortality of workers showed an upward trend. However, for the root extracts, no concentration achieved 100% mortality by day 6, with progressive mortality rates correlating with dosage (Figure 2a). For the stem extracts at 25 mg/mL, 100% mortality was observed by day 6, while 12.5 mg/mL and 6.25 mg/mL yielded 90% and 91.7% mortality, respectively (*p* < 0.05 vs. control) (Figure 2b). On the other hand, the ethanol extracts of the leaves had the best insecticidal effect. Leaf extracts at concentrations ≥ 6.25 mg/mL induced complete mortality (100%) by day 6, with significant divergence from controls (*p* < 0.05) (Figure 2c).

The concentrations of ethanol leaf extracts at 3.125 mg/mL and 1.5625 mg/mL were selected together with control to explore their effects on the behavior of workers, and the results showed that at both concentrations, the aggression rate reduced to 65% and 46.7% by day 5, respectively (vs. control: 78.3%; *p* < 0.05) (Figure 2d). The climbing ability declined to 63.33% and 48.33%, respectively (Figure 2e). Moreover, the grasping ability decreased from 91.3% (day 1) to 60% (3.125 mg/mL) and 43.3% (1.56 mg/mL) by day 5, respectively (Figure 2f).

As summarized in Table 1, insecticidal activity varied markedly by plant tissue, with leaf extracts showing the highest lethality and behavioral disruption.

### 4.3. Toxicity and Behavioral Effects of Safranal and Dihydrocoumarin on S. invicta

**Figure 3:**
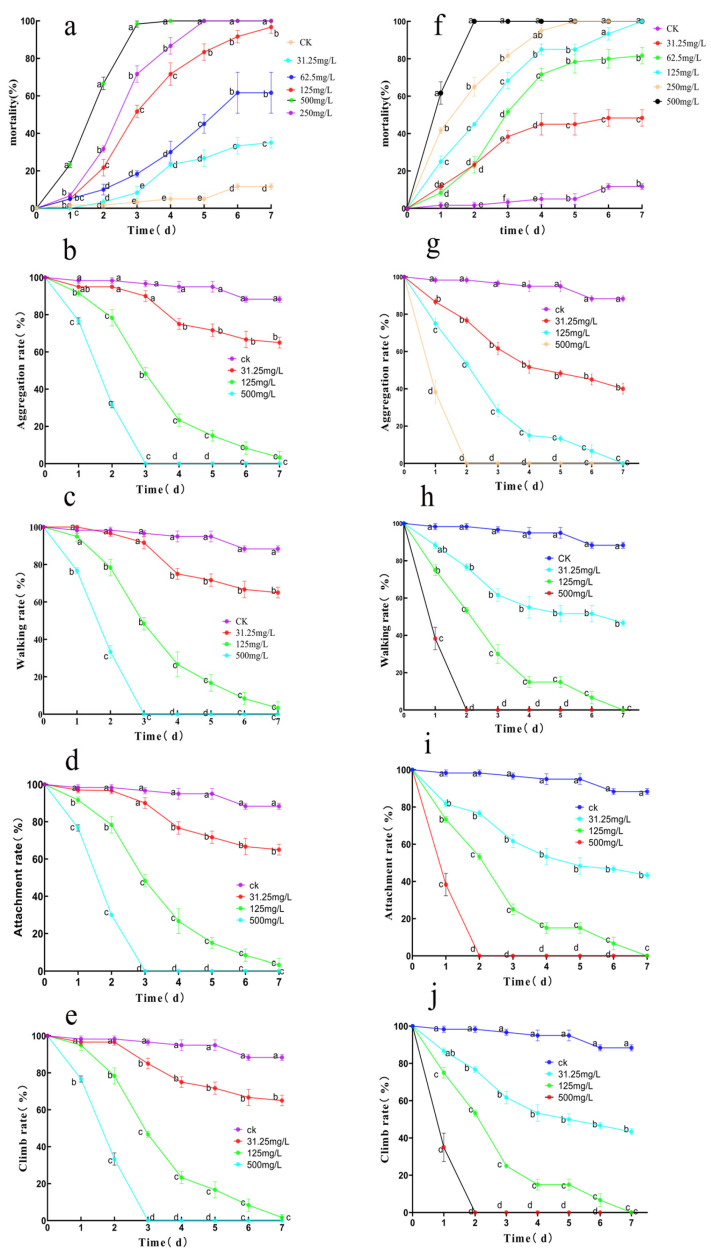
Toxicity and behavioral effects of Safranal and Dihydrocoumarin on *Solenopsis invicta*. (**a**) Lethal effects of different concentrations of Dihydrocoumarin on *Solenopsis invicta*. (**f**) Lethal effects of different concentrations of Safranal on *Solenopsis invicta*. (**b**–**e**,**g**–**j**) Effect of different concentrations of Safranal and Dihydrocoumarin on the grasping capacity, walking ability, aggression, and climbing ability of *Solenopsis invicta*. Data are presented as mean ± standard error (S.E.). Different letters above bars indicate significant differences in mortality due to concentration effects within the treatment at *p* < 0.05 level.

Safranal and dihydrocoumarin exhibited dose-dependent insecticidal activity and behavioral inhibition against *S. invicta* workers (Figure 3). Dihydrocoumarin induced 100% mortality by day 2 in *S. invicta* workers at 500 mg/mL, while by day 7, concentrations of 500, 250, 125, 62.5, and 31.25 mg/mL induced mortality rates of 100%, 100%, 100%, 81.7%, and 48.3%, respectively (*p* < 0.05 vs. control: 11.7%) (Figure 3a). Safranal attained 100% mortality at 500 mg/mL by day 3. On day 7, concentrations ≥125 mg/mL caused >96.7% mortality (*p* < 0.05 vs. control) (Figure 3f).

At concentration of 500 mg/mL, 125 mg/mL, and 31.25 mg/mL of safranal (Figure 3g–j) and dihydrocoumarin (Figure 3b–e), the aggression, grasping, walking, and climbing abilities of workers were significantly inhibited, and the inhibitory effect increased with an increase in concentration. Both compounds at high concentration (500 mg/mL) induced complete inhibition (100%) of all behaviors by day 3. On the 7th day, near-complete inhibition (>95%) at 125 mg/mL and partial suppression (<80%) at 31.25 mg/mL were observed.

Both safranal and dihydrocoumarin had significant insecticidal activity against workers, while dihydrocoumarin demonstrated faster lethality than safranal at 48 h, as summarized in Table 2.

### 4.4. Metabolic Profiling of S. invicta Against Safranal and Dihydrocoumarin

#### 4.4.1. Principal Component Analysis

**Figure 4:**
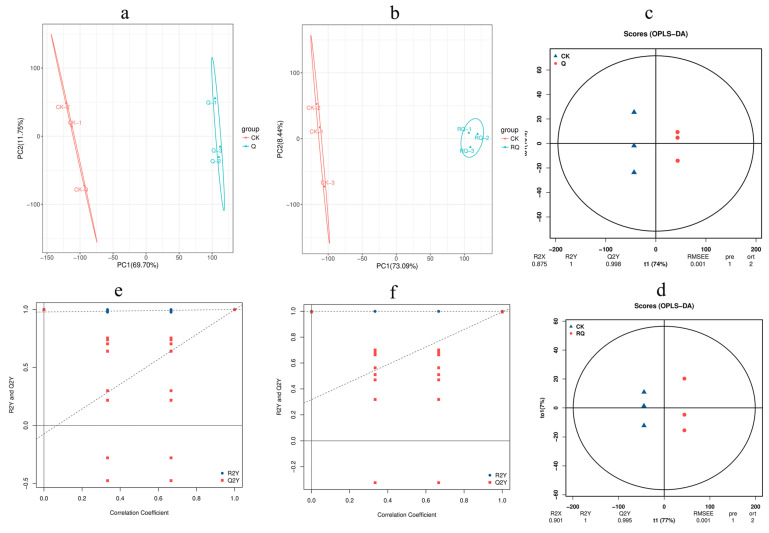
Principal component analysis and orthogonal partial least squares discriminant analysis. (**a**,**c**,**e**) CK_vs_Q, (**b**,**d**,**f**) CK_vs_Q.

The principal component analysis (PCA) score plot can reflect the degree of similarity of samples. The more clustered samples depict a stronger similarity degree of the samples [19]. The sample groups were analyzed by multivariate statistical PCA. The contribution rate of principal component 1 of CK (control) and safranal (Q) was 69.7%, the contribution rate of principal component 2 was 11.75%, and the cumulative variance contribution rate of the two was 81.45% (Figure 4a). The contribution rate of principal component 1 of RQ (dihydrocoumarin) was 73.09%, the contribution rate of principal component 2 was 8.44%, and the cumulative contribution rate of the two was 81.53% (Figure 4b). CK and Q and CK and RQ were clearly divided into two groups, which were basically effectively distinguished, indicating that there were significant differences between the different groups.

#### 4.4.2. Orthogonal Partial Least Squares Discriminant Analysis

Orthogonal partial least squares discriminant (OPLS-DA) can filter out information irrelevant to classification and accurately analyze the differences between different groups. OPLS-DA was used to analyze the mass spectrometry data, and it was found that the CK and Q, CK and RQ, two groups of samples, were distributed on the left and right sides of the confidence interval, respectively, with obvious differentiation effects, indicating that there were significant differences between the groups (Figure 4a,b). The permutation test diagram of the OPLS-DA model shows that the slopes of the Q2Y fitted regression lines of CK and Q, CK and RQ, and the two groups are all positive, indicating that the model is meaningful. The blue points are generally located above the red points, indicating that the independence of the sets is good (Figure 4e,f).

#### 4.4.3. Metabolic Mechanism Analysis

**Figure 5:**
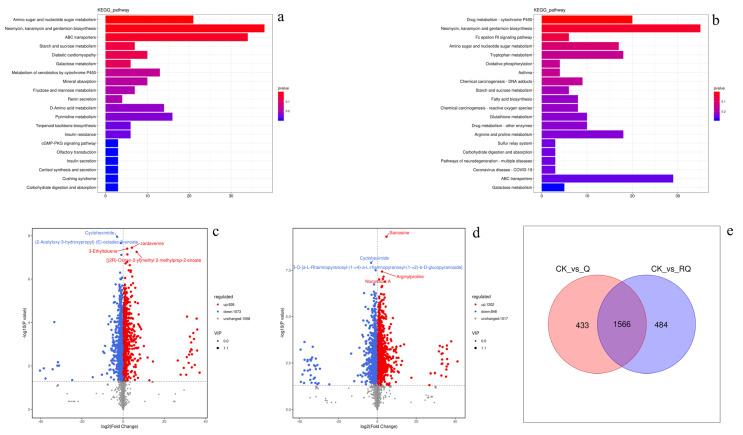
Differential metabolite analysis. (**a**) CK_vs_Q KEGG enriched pathway, (**b**) CK_vs_RQ KEGG enriched pathway, (**c**) CK_vs_Q differential metabolite statistical volcano plot, (**d**) CK_vs_RQ differential metabolite statistical volcano plot, and (**e**) CK_vs_Q and CK_vs_RQ Wayne analysis.

Differential metabolites were screened according to fold change ≥ 1 and *p* value < 0.05. The volcano plot showed that 3067 metabolites were identified in both CK_vs_Q and CK_vs_RQ. There were 926 significantly upregulated metabolites and 1073 significantly downregulated metabolites in CK_vs_Q (Figure 5c), and there were 1202 significantly upregulated metabolites and 848 significantly downregulated metabolites in CK_vs_RQ (Figure 5d). Based on the Venn diagram, the intersection and union of the differential metabolites between the groups were compared and analyzed. There were 433 unique metabolites in CK_vs_Q, 484 unique metabolites in CK_vs_RQ, and 1566 metabolites shared by the two comparison groups (Figure 5e).

**Figure 6:**
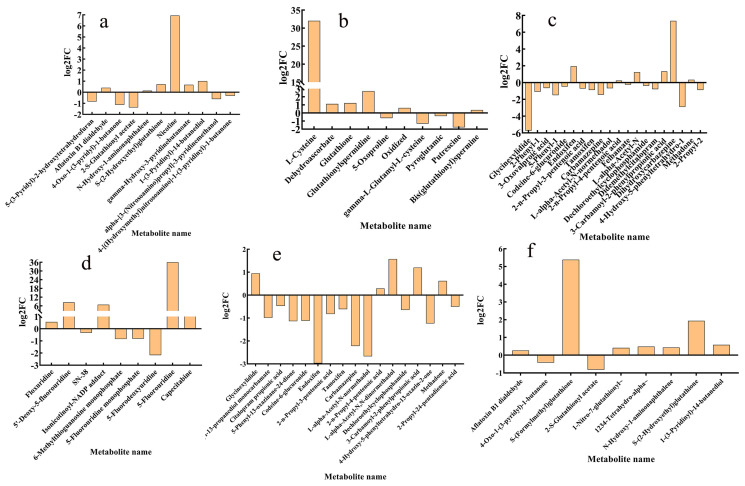
Analysis of gene expression related to detoxification metabolism of workers. (**a**) CK_vs_Q Metabolism of xenobiotics by cytochrome P450, (**b**) CK_vs_Q Glutathione metabolism, (**c**) CK_vs_Q drug metabolism—cytochrome P450, (**d**) CK_vs_Q drug metabolism—other enzymes, (**e**) CK_vs_RQ drug metabolism—cytochrome P450, and (**f**) CK_vs_RQ metabolism of xenobiotics by cytochrome P450.

The KEGG (Kyoto Encyclopedia of Genes and Genomes) database helps researchers study genes, expression information, and metabolite content as a whole network. The KEGG database is a collection of small molecules, biopolymers, and other chemicals related to biological systems included in the KEGG database and provides annotations of these substances in the KEGG pathway database. The KEGG results of the differential metabolite enrichment bar chart showed that the metabolic pathways involved in the detoxification metabolism in insects by CK_vs_Q include drug metabolism—cytochrome P450, glutathione metabolism, and drug metabolism—other enzymes (Figure 6a); those of CK_vs_RQ include metabolism of xenobiotics by cytochrome P-450 (Figure 5b). The metabolisms that CK_vs_Q and CK_vs_RQ participate most in include various amino acid metabolisms, sugar metabolism, and starch metabolism (Figure 5a,b).

After ingesting or coming into contact with toxic substances, red fire ants detoxify them through metabolic pathways in their bodies. After feeding the red fire ants with honey water containing safranal and dihydrocoumarin, the metabolic pathways in their bodies changed significantly. Most of the metabolites enriched in KEGG in CK_vs_Q and related to metabolism of xenobiotics by cytochrome P-450, glutathione metabolism, drug metabolism—cytochrome P-450, and drug metabolism—other enzymes showed significant decreases compared with the control group: CK_vs_RQ. There were 26 differential metabolites in the drug metabolism—cytochrome P-450 pathway, among which 12 metabolites decreased significantly, the most obvious of which were Endoxifen, Carbamazepine, and L-alpha-Acetyl-N-normethadol. Of the nine metabolites of xenobiotics by cytochrome P-450, seven increased in content, while two decreased in content (Figure 6).

**Figure 7:**
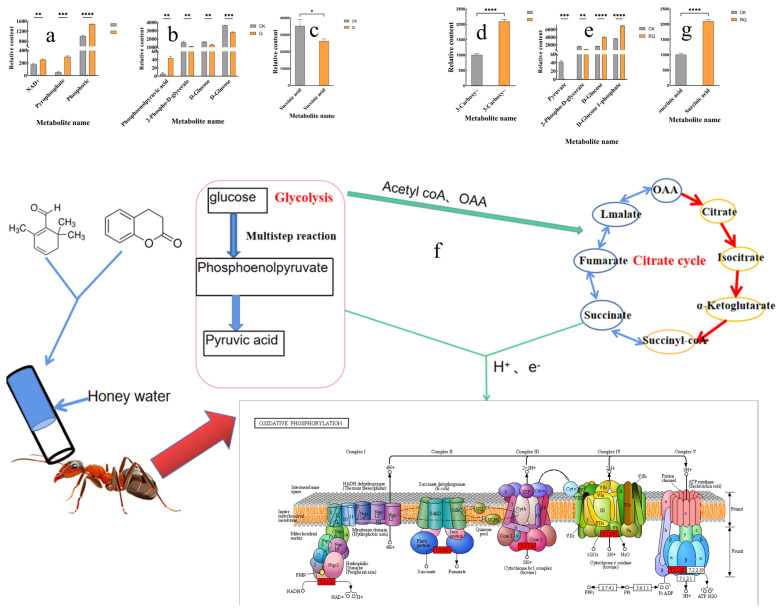
Energy metabolism analysis after treatment with two compounds. (**a**) CK_vs_Q, oxidative phosphorylation; (**b**) CK_vs_Q, glycolysis; (**c**) CK_vs_Q, citrate cycle; (**d**) CK_vs_RQ, oxidative phosphorylation; (**e**) CK_vs_RQ, glycolysis; (**g**) energy cycle diagram; and (**f**) CK_vs_RQ, citrate cycle, 3-Carbamoyl-:3-Carbamoyl-2-phenylpropionic acid. Note: * represents a *p*-value less than 0.05, ** represent a *p*-value less than 0.01, *** represent a *p*-value less than 0.001, and **** represent a *p*-value less than 0.0001.

After feeding honey water containing safranal and dihydrocoumarin, the main energy metabolism pathways (glycolysis, citrate cycle, and oxidative phosphorylation) in the bodies of workers were disrupted. The three major energy metabolisms each produce a large amount of ATP, and at the same time, they are connected to each other. Glycolysis provides acetyl COA and OAA for the citrate cycle, and glycolysis and the citrate cycle provide H^+^ and e^−^ for oxidative phosphorylation to form ATP. (Figure 7f) After the ants consumed honey water containing dihydrocoumarin, the difference in substances in the citrate cycle and glycolysis showed an overall downward trend, while the difference in substances in oxidative phosphorylation increased, and the material circulation and energy metabolism were abnormal (Figure 7a–c). After consuming honey water containing dihydrocoumarin, the difference in substances in glycolysis, citrate cycle, and oxidative phosphorylation increased, reflecting the multi-target interference of dihydrocoumarin on energy metabolism (Figure 7d–f).

### 4.5. Effects of Safranal and Dihydrocoumarin on Activities of Four Enzymes of S. invicta

**Figure 8:**
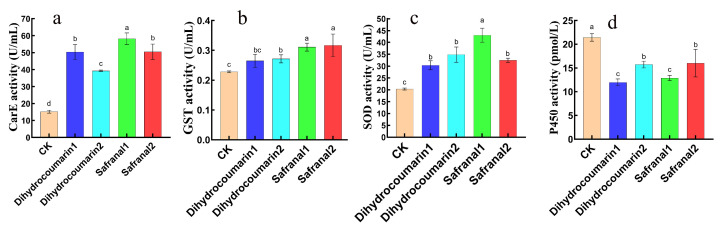
Effects of Safranal and Dihydrocoumarin on enzyme activity of *Solenopsis invicta*. Data are presented as mean ± standard error (S.E.). Different letters above bars indicate significant differences in mortality due to concentration effects within treatment at *p* < 0.05 level. (**a**) Carboxylesterase; (**b**) glutathione S-transferase; (**c**) superoxide dismutase; and (**d**) insect cytochrome P-450 enzyme 1: LC_50_ 2: double LC_50_.

Safranal and Dihydrocoumarin had significant effects on superoxide dismutase, insect cytochrome P-450 enzymes, glutathione S-transferase, and carboxylesterase of workers. The carboxylesterase activity after treatment with LC_50_ and double LC_50_ concentrations of Safranal and Dihydrocoumarin was significantly increased compared with CK (*p* < 0.05). The enzyme activity after double LC_50_ treatment was significantly higher than CK but lower than LC_50_ (*p* < 0.05), showing a significant difference (Figure 8a), and the activity of glutathione S-transferase showed an increasing trend with the increase in compound concentration and was significantly higher than that of CK (*p* < 0.05) (Figure 8b). The activity of superoxide dismutase increased significantly after treatment with dihydrocoumarin at LC_50_ and double LC_50_ concentrations (*p* < 0.05), and there was no significant difference between LC_50_ and double LC_50_. Although the activity of superoxide dismutase after treatment with safranal was significantly higher than that of CK (*p* < 0.05), double LC_50_ concentration treatment was significantly lower than the LC_50_ treatment (*p* < 0.05) (Figure 8c); the insect cytochrome P-450 enzymes were significantly lower than CK after Safranal and Dihydrocoumarin LC_50_ and double LC_50_ concentration treatment (*p* < 0.05), However, after treatment with double LC_50_ concentration, there was a significant upward trend relative to LC_50_ (*p* < 0.05) (Figure 8d).

## 5. Discussion

Extracts derived from botanical sources have long been recognized for their insecticidal potential against invasive ants, particularly *S. invicta*. Prior studies, for instance, demonstrated the efficacy of Datura metel root, stem, and leaf extracts in inducing mortality and impairing locomotion behaviors (e.g., climbing, aggression) in red imported fire ants [30]. Similarly, ethanolic extracts from *Lantana camara*, *Camptotheca acuminata*, and *Ligusticum chuanxiong* exhibited concentration-dependent lethality, corroborating the broader applicability of plant-derived compounds in ant management [31]. The present study aligns with these findings, revealing that *A. adenophora* leaf and stem extracts induce significant mortality (up to 100% at 25 mg/mL) and behavioral inhibition (e.g., aggression, grasping) in *S. invicta* workers, while root extracts showed comparatively reduced efficacy (<50% mortality). The observed behavioral disruptions—declines in aggression (46.7%), climbing (48.3%), and grasping (43.3%) at sub-lethal doses—parallel patterns reported for pyrethrins and nicotine sulfate, suggesting conserved neurotoxic pathways among botanicals [30].

We used a metabolomics approach to preliminarily explore the possible main bioactive compounds from the *A. adenophora*. One or more of safranal, 3,4-Dihydrocoumarin, 7-hydroxycoumarin, demethylsuberosin, bergaptol, methoxsalen, scoparone, monacolin L acid, and monacolin J acid in the biosynthesis of other secondary metabolites may be the substances that cause the death of workers. It has been reported that colchicine in *Colchicum dacchini* has toxicity and behavioral inhibition to *S. invicta* workers, which is consistent with our experimental results. However, the absence of quantitative profiling for safranal and dihydrocoumarin in *A. adenophora* tissues represents a critical limitation, mirroring gaps in earlier studies, such as colchicine quantification in *Colchicum dacchini* [29]. Notably, safranal’s documented anti-inflammatory roles in mammalian systems contrast with its arthropod-specific toxicity observed here, suggesting species-dependent mechanistic divergence that warrants further investigation [32]. Future work should prioritize dose– and time–response assays using purified compounds to isolate their individual efficacies, alongside field trials to assess ecological persistence and non-target impacts.

Regarding the detoxification and other metabolic enzyme changes in insect bodies in response to the ingestion of any toxic substances, studies have shown that superoxide dismutase (SOD) in forest tent caterpillars and gypsy moths changes in different ways after pesticidal treatment [33]. We investigated the changes in SOD, insect cytochrome P450 enzymes, glutathione S-transferase (GST), and carboxylesterase (CarE) in *S. invicta* workers after they ingested honey water containing safranal and dihydrocoumarin (Figure 8). After the ants fed on honey water containing safranal and dihydrocoumarin, the enzyme activity of CarE was significantly higher than that of CK at the LC_50_ and 2LC_50_ concentrations, but the activity was lower at high concentrations than at low concentrations. It is possible that the ants produced an adaptive stress response at low concentrations, and at high concentrations, the compounds directly inhibited CarE, reducing the detoxification ability of ants to insecticides. After feeding with the two compounds, the GST enzyme activity showed an increasing trend. Safranal, as an electrophilic compound, may be catalyzed by GST to bind to GSH, consume GSH, and temporarily increase GST activity. Dihydrocoumarin may be catalyzed by GST to bind to GSH due to substrate metabolism, resulting in a temporary increase in GST activity. For SOD, Safranal has antioxidant properties, which may directly enhance antioxidant capacity by scavenging free radicals or indirectly upregulate SOD expression by activating the Nrf2/ARE pathway. Dihydrocoumarin has potential toxicity, and coumarin derivatives may induce oxidative stress by generating toxic intermediates (such as epoxides) through metabolism, thereby upregulating SOD activity. However, the changes in P450 enzyme activity are opposite to those of the above three enzymes, and the enzyme activity is significantly lower than that of the control, which may be caused by the treatment time and dosage.

Changes in metabolic pathways after insects ingest toxic compounds are a complex and important biological phenomenon [34]. After feeding on honey water containing safranal and dihydrocoumarin, the metabolic pathways in the bodies of workers changed significantly, and energy metabolism was disrupted. After consuming dihydrocoumarin, glycolysis may be blocked, resulting in a decrease in pyruvate production. The decrease in the rate of acetyl-CoA entering the mitochondria directly limits the initiation of the TCA cycle. The increase in oxidative phosphorylation-related substances may be due to dihydrocoumarin inhibiting the complexes of the mitochondrial ETC (such as complex I or II), resulting in blocked electron transfer. Electrons accumulate and leak in the ETC, reacting with oxygen molecules to generate reactive oxygen such as superoxide anions, which is manifested as an increase in the level of oxidative phosphorylation-related free radicals. Enhanced sugar metabolism leads to increased pyruvate production. If the mitochondrial membrane has not been completely damaged, pyruvate can be converted into acetyl-CoA and enter the TCA cycle, resulting in the accumulation of early intermediates, such as citric acid and isocitric acid. Dihydrocoumarin may inhibit ETC complex III (cytochrome c reductase) or complex IV (cytochrome c oxidase), resulting in blocked electron transfer. Electrons accumulate on the inner membrane of the mitochondria and react with oxygen molecules to generate superoxide anions and hydrogen peroxide, which is manifested as an increase in the level of oxidative phosphorylation-related free radicals. Metabolism of xenobiotics by cytochrome P-450, glutathione metabolism, drug metabolism—cytochrome P-450, and drug metabolism—other enzymes were significantly enriched. This may be directly related to the ants’ active resistance to the toxic effects of the two compounds.

## 6. Conclusions

Safranal and dihydrocumarin in the secondary metabolites of *A. adenophora* can affect the citrate cycle, glycolysis, and oxidative phosphorylation of workers, interfere with normal energy metabolism and enzyme activity, inhibit their walking and attacking abilities, etc., as well as have a good control effect on *S. invicta*.

## Figures and Tables

**Table 1:** The 50% lethal concentration of *Ageratina adenophora* ethanol extracts against *Solenopsis invicta* at 48 h.

Different Parts Extracts	Regression Equation	50% Lethal Concentration (mg/mL)	95% Fiducial Limits	*p*-Value
Leaf	Y = 0.008X − 1.366	166.253	144.867~194.392	*p* < 0.05
Stems	Y = 0.008X − 1.429	188.256	141.716~284.943	*p* < 0.05
Root	Y = 0.006X − 1.851	331.847	270.168~452.323	*p* < 0.05

**Table 2:** The 50% lethal concentration of two compounds against *Solenopsis invicta*.

Metabolite Name	Regression Equation	50% Lethal Concentration (mg/L)	95% Fiducial Limits	*p*-Value
Safranal	Y = 3.44X − 8.759	349.042	282.542~461.604	*p* < 0.05
Dihydrocoumarin	Y = 3.232X − 6.701	118.336	16.541~673.671	*p* < 0.05

## Data Availability

The original contributions presented in this study are included in the article. Further inquiries can be directed to the corresponding author.

## Abbreviations

The following abbreviations are used in this manuscript:

LC-MS	Liquid chromatography–mass spectrometry
LC_50_	50% lethal concentration
UPLC	Ultra-high-performance liquid chromatography
S.E.	Standard error
PCA	Principal component analysis
KEGG	Kyoto Encyclopedia of Genes and Genomes
SOD	Superoxide dismutase
GST	Glutathione S-transferase
CarE	Carboxylesterase
QC	Quality control
ESI	Electrospray ionization
GSII	Gas II
CUR	Curtain gas
DP	Declustering potential
CE	Collision energy
Q	Safranal
RQ	Dihydrocoumarin
ANOVA	One-way analysis of variance
PMDB	Plant Metabolome Database
TCA	Tricarboxylic acid cycle
